# Silencing TRAIP suppresses cell proliferation and migration/invasion of triple negative breast cancer via RB-E2F signaling and EMT

**DOI:** 10.1038/s41417-022-00517-7

**Published:** 2022-09-05

**Authors:** Yan Zheng, Huiqing Jia, Ping Wang, Litong Liu, Zhaoxv Chen, Xiaoming Xing, Jin Wang, Xiaohua Tan, Chengqin Wang

**Affiliations:** 1 Department of Pathology, School of Basic Medicine, Qingdao University, Qingdao, Shandong 266021 China; 2 Department of Pathology, The Affiliated Hospital of Qingdao University, Qingdao, Shandong 266000 China

**Keywords:** Breast cancer, Gene expression, Metastasis, Biomarkers, Oncogenes

## Abstract

TRAIP, as a 53 kDa E3 ubiquitin protein ligase, is involved in various cellular processes and closely related to the occurrence and development of tumors. At present, few studies on the relationship between TRAIP and triple negative breast cancer (TNBC) were reported. Bioinformatic analysis and Western blot, immunohistochemistry (IHC), CCK-8, colony formation, flow cytometry, wound healing, Transwell, and dual-luciferase reporter assays were performed, and xenograft mouse models were established to explore the role of TRAIP in TNBC. This study showed that the expression of TRAIP protein was upregulated in TNBC tissues and cell lines. Silencing of TRAIP significantly inhibited the proliferation, migration, and invasion of TNBC cells, whereas opposite results were observed in the TRAIP overexpression. In addition, TRAIP regulated cell proliferation, migration, and invasion through RB-E2F signaling and epithelial mesenchymal transformation (EMT). MiR-590-3p directly targeted the TRAIP 3′-UTR, and its expression were lower in TNBC tissues. Its mimic significantly downregulated the expression of TRAIP and subsequently suppressed cell proliferation, migration, and invasion. Rescue experiments indicated that TRAIP silencing reversed the promotion of miR-590-3p inhibitor on cell proliferation, migration, and invasion. TRAIP overexpression could also reverse the inhibition of miR-590-3p mimic on tumorigenesis. Finally, TRAIP knockdown significantly inhibited tumor growth and metastasis in animal experiments. In conclusion, TRAIP is an oncogene that influences the proliferation, migration, and invasion of TNBC cells through RB-E2F signaling and EMT. Therefore, TRAIP may be a potential therapeutic target for TNBC.

## Background

Breast cancer is one of the most common malignant tumors in the world and the main cause of death from cancer in women [1, 2]. Triple negative breast cancer (TNBC), a subtype of breast cancer, refers to breast cancer with negative expression levels of human epidermal growth factor receptor 2 (HER2), estrogen receptor (ER), and progesterone receptor (PR) [3]. Patients with TNBC could not benefit from endocrine therapy and HER2 targeted therapy, resulting in higher recurrence, metastasis rates, and mortality [4, 5]. Therefore, study on new therapeutic targets and targeted drugs for TNBC has become a research hotspot locally and internationally.

Proliferative activity is the basis and prerequisite of tumor migration and invasion. Studies have shown that RB-E2F signaling pathway played an important role in tumor cell proliferation [6]. Gene mutations of this pathway are widespread in TNBC, including CCND1 (encoding Cyclin D1) and CCNE1 (encoding Cyclin E1) gene amplification and RB gene deletion [7]. RB-E2F signaling is also involved in cell migration, angiogenesis, and epithelial mesenchymal transformation (EMT) [8, 9]. Knockdown of E2F2 resulted in enhanced migration of TNBC cell line MDA-MB-231 and increased lung metastases in mice [10]. EMT is known to be one of the important mechanisms of tumor metastasis, characterized by epithelial cells losing cell connections and acquiring mesenchymal properties, such as motility and invasiveness [11, 12]. Dongre et al. showed that the expression of epithelial marker was decreased, while mesenchymal marker and nuclear transcription factors were upregulated [13]. Some scholars found the expression of EMT-related genes in breast cancer cell lines inactivated by RB1 [14], and E-cadherin, Snail, and Twist genes were highly expressed in TNBC [15].

The tumor necrosis factor (TNF) receptor-related factor (TRAF) interacting protein (TRAIP) is a 53 kDa E3 ubiquitin protein ligase containing three domains: the RING domain near the N-terminal, followed by the putative coiled-coil domain and the leucine zipper domain [16, 17]. TRAIP was reported to be involved in various cellular processes, including cell proliferation, DNA damage response, mitosis, and embryonic development [18–21]. TRAIP was located near the mitotic chromosomes and regulated the mitosis process through spindle assembly checkpoints [22]. In particular, it played a new role in the mitosis process through the isotopic dimerization of its coiled-coil domain [23]. Park et al. created TRAIP-deficient mice and found that the mice shortly died due to early embryonic development [24]. Silencing of TRAIP resulted in a strong inhibition of human keratinocyte cell proliferation and cell cycle arrest [25]. In addition, the inactivation of TRAIP caused serious damage to and scarcity of nucleotides, thereby endangering genome stability and revealing that TRAIP is a component of the mammalian replication stress response network [26]. All of the above data suggested that TRAIP palyed an extremely vital role in the cell process and laid a cytological foundation for the occurrence and development of tumors. However, up to now, few studies focused on the relation between TRAIP and TNBC.

The first microRNA (miRNA) was identified in 1993 as a small RNA transcribed from the *Caenorhabditis elegans* lin-4 locus [27, 28]. The prediction results of a bioinformatic software showed that miR-590-3p was the upstream molecule of TRAIP. MiR‐590‐3p could also inhibit EMT, cell migration, and cell invasion in breast cancer [29]. However, the detailed association among miR-590-3p, TRAIP, RB-E2F signaling, and EMT in TNBC remained unclear.

In this study, the expression and role of TRAIP in TNBC and cell line proliferation, migration, and invasion were investigated. In addition, the mechanism of TRAIP regulating the proliferation and migration/invasion of TNBC cells was further explored by analyzing the relationship among miR-590-3p, TRAIP, RB-E2F signaling, and EMT. This study may provide theoretical basis for seeking a new target of anti-TNBC therapy.

## Methods

### Bioinformatic analysis

UALCAN (http://ualcan.path.uab.edu/) is an online open access platform that could be used to analyze the relative transcription levels and clinicopathological characteristics between cancer tissues and paired normal tissues [30]. The target gene “TRAIP” was entered on the homepage of the website, “breast invasive carcinoma” was selected, and the differential expression of the target gene in breast cancer tissues and normal tissues was obtained. The differential expression of TRAIP from a sample type (normal/primary tumor) and a breast cancer subtype (luminal, HER2 + , and triple negative) was analyzed. Meanwhile, Kaplan–Meier plotter was used evaluate the impact of 54,000 genes on the survival rate of 21 cancer types. Hazard ratio (HR), 95% confidence interval (CI), and logarithmic P value were also automatically calculated and displayed on the web page. Patients were divided into high-expression and low-expression TRAIP according to the automatic best threshold that all possible cutoff values between the lower and upper quartiles are computed. A log-rank P value < 0.05 was considered statistically significant.

### Tissue samples and cell lines

Seventy-five female patients with primary TNBC treated at the Affiliated Hospital of Qingdao University between 2013 and 2015 participated in this study. All patients did not receive chemotherapy nor radiotherapy before surgery, and the related clinical information (Table 1) was obtained from all patients with written consent. This study was reviewed and approved by the Institutional Medical Ethics Committee of the Qingdao University Affiliated Hospital.

**Table 1:** Association between TRAIP in triple negative breast cancer (TNBC) and patient characteristics.

Variables	*n*	Mean±SD	*P* value
Age			
≤60 years	44	5.818 ± 0.815	0.504
>60 years	31	5.581 ± 0.765	
Tumor size (cm)			
≤2	34	5.706 ± 0.799	0.985
>2	41	5.732 ± 0.807	
Tumor grade			
II	25	5.920 ± 0.812	0.499
III	50	5.620 ± 0.780	
Carcinoma	75	5.720 ± 0.798	<0.001*
Adjacent tissues	75	4.467 ± 0.622	
Lymph node metastasis			
Negative	55	5.564 ± 0.788	0.166
Positive	20	6.150 ± 0.671	
Vascular invasion			
Presence	10	6.400 ± 0.700	0.096
Absence	65	5.615 ± 0.764	
Primary tumor	20	6.150 ± 0.671	0.001*
Metastatic tumor in lymph nodes	20	7.050 ± 0.605	
Tumor recurrence			
Positive	22	6.181 ± 0.665	0.038*
Negative	53	5.528 ± 0.775	

^*^Significant at <0.05.

Human breast cancer cell lines MCF-7, MDA-MB-231, MDA-MB-468, and BT-549 were routinely cultured in DMEM medium containing 10% fetal bovine serum and 1% penicillin and streptomycin at an incubator (37°C and 5% CO_2_). The cells in logarithmic growth phase were taken for the subsequent experiment.

### Immunohistochemistry (IHC) analysis

The procedure of IHC staining was performed as described previously [31]. Anti-TRAIP (dilution at 1:300, 4°C, overnight) and sheep anti-rabbit (abcam, dilution at 1:100, 37°C, 30 min) were incubated. Sections were stained with diaminobenzidine and counterstained with hematoxylin. Phosphate-buffered saline (PBS) was used as negative control. The criteria for the interpretation of immunohistochemistry were scored in accordance with staining intensity and tumor cell positive ratio. The sum of staining intensity (0, none; 1, weak; 2, intermediate; and 3, strong) and positive tumor cell proportion (0, none;1, < 1/100; 2, 1/100–1/10; 3, 1/10–1/3; 4, 1/3–2/3; and 5, > 2/3) was regarded as the total score, which ranged from 0 to 8.

### Vector construction and cell transfections

LV3-hsa-TRAIP-318\543\131 (ShTRAIP-1, ShTRAIP-2, and ShTRAIP-3) and LV5-hsa-TRAIP-homo (OETRAIP) were constructed by lentiviral vectors (GenePharma, Shanghai, China). Negative control was also constructed with LV3 (ShCtrl) and LV5 (NC) empty lentiviral separately. MDA-MB-231 and MDA-MB-468 cells were transfected with the ShTRAIP-1/2/3 vector to silence TRAIP and LV3 empty lentiviral as ShCtrl. BT-549 cell was transfected with LV5-hsa-TRAIP-homo (OETRAIP) to overexpress TRAIP and LV5 empty lentiviral as NC. TNBC cells were transfected using lentiviral vectors at an appropriate multiplicity of infection (MOI) when cells grew to 50%–70% confluence. Stable transfected cells were screened by puromycin in accordance with protocols. MiR‐590‐3p mimic and miR‐590‐3p inhibitor, negative control for miRNA mimic, and negative control for miRNA inhibitor were purchased from GenePharma. The 3′-UTR segment of wild-type TRAIP mRNA, which possessed the binding site for miR-590-3p, was amplified from the DNA of 293 T cells and cloned into the luciferase reporter vector pGL3cM (Tsingke, Beijing, China). All sequences are listed in Table 2.

**Table 2:** Sequences of TRAIP, miR-590-3p and their negative control.

Name	Sequence (5’−3’)
LV3-TRAIP-homo-318	GGAGGAGAATGTCTTGGATGC
LV3-TRAIP-homo-543	GCAGCAGGATGAGACCAAACA
LV3-TRAIP-homo-131	GCACTATCTGCTCCGACTTCT
LV3NC	TTCTCCGAACGTGTCACGT
Has-miR-590-3p mimics	UAAUUUUAUGUAUAAGCUAGU
	UAGCUUAUACAUAAAAUUAUU
Negative control mimics	UUCUCCGAACGUGUCACGUTT
	ACGUGACACGUUCGGAGAATT
Has-miR-590-3p inhibitor	ACUAGCUUAUACAUAAAAUUA
Negative control inhibitor	CAGUACUUUUGUGUAGUACAA

### Western blot analysis

Protein preparation and Western blot assay were performed as described previously [31]. The antibodies used were as follows: anti-TRAIP (ProteinTech, dilution at 1:1000), anti-β-actin (ProteinTech, dilution at 1:4000), anti-RB, anti-P-RB, anti-E2F1, anti-Cyclin D1, anti-Cyclin E1, anti-P21, anti-MMP9, anti-Twist, anti-Slug, anti-E-cad (abcam, all at dilution 1:1000), anti-MMP2, and anti-Vimentin (abcam, all at dilution at 1:500).

### Cell proliferation analysis

A CCK-8 kit (Dojindo, Shanghai, China) was used to measure the proliferation of TNBC cells. A total of 2000 cells at a volume of 100 μL per well were cultured, with six replicate wells in a 96-well plate. Then, the CCK-8 reagent (10 μL) was added to generate a working solution and incubated for 2 h. The assay was performed at days 1–5. For colony formation assay, 2 × 10^3^ cells were seeded in a six-well plate and cultured for approximately 10 days at the described condition. Then, the colonies were washed with PBS, fixed with methanol, and dyed with crystal violet. Finally, they were counted using Image software.

### Cell cycle analysis by flow cytometer (FCM)

Cells were washed with ice-cold PBS twice and harvested by trypsinization without EDTA in six-well plates. After centrifugation was performed for 5 min, the cells were washed with ice-cold PBS and fixed with 70% ethanol overnight at 4°C. RNaseA (20 μg/mL) and propidium iodine (50 µg/mL) were added to the cells for 30 min in the dark. The stained cells were then analyzed with an FCM (Beckman Coulter).

### Transwell assays

In the invasion experiment, 6 × 10^4^ cells per well were suspended in serum-free medium and loaded into the upper compartment of a chamber coated with Matrigel (BD Biosciences). After 24 h of incubation at 37°C, the invasive cells were migrated through the Matrigel to a medium containing 20% serum in the lower compartment and stained with 0.5% crystal violet. The number of invading cells in five random microscope fields (100×) was counted. For migration analysis, 4 × 10^4^ cells were seeded in the upper chamber that was not coated with Matrigel and then measured in accordance with the invasive assay.

### Wound healing assay

The wounds were caused by a 200 μL-sterile yellow tip when the cells reached 90% confluence in six-well plates. The cells were gently washed with PBS to remove the shed cells, and serum-free medium was added for further culture. Subsequently, the wounds were photographed and calculated under the microscope after 0 and 24 h.

### Dual-luciferase reporter assay

Luciferase reporter assays were conducted to demonstrate whether miR-590-3p was a direct target of TRAIP. Wild-type (WT) and mutant-type (MUT) TRAIP 3′-UTRs were transfected into 293 T cell with synthetic miR-590-3p mimic or NC mimic. The cells were lysed, and the activities of Renilla luciferase and Firefly luciferase were detected by a dual-luciferase reporter system (Promega, USA) following the provided protocols. Data were presented as the ratio of experimental (Renilla) luciferase to control (Firefly) luciferase.

### Xenograft assays in nude mice

The animal study was approved by the Animal Ethics Committee of Qingdao University, China. In particular, 1 × 10^7^ ShCtrl and ShTRAIP-1 MDA-MB-231 cells were subcutaneously implanted into 5-week-old female BABL/c nude mice, with five mice in each group. The size and weight of tumors were recorded every 7 days, and tumor volume was measured using the following formula: volume (mm^3^) = (width^2^×length)/2. In addition, 10 BABL/c mice were randomly divided into two groups (five in each group) and injected with ShCtrl and ShTRAIP-1 MDA-MB-231 cells (1 × 10^6^) via tail vein. Six weeks later, the mice were euthanized. The whole lung tissue of each mouse was sectioned and stained with hematoxylin and eosin (H&E), and metastatic nodules were counted in high-power fields under a microscope.

### Statistical analyses

All dates were presented as the mean ± standard deviation, and each experiment was performed at least three times. Statistical analyses were performed using Graphpad 8.0 software. Student’s *t*-test (two tailed) and ANOVA were utilized to detect differences between two groups or more than two groups. Chi-square test was used to estimate the correlation between TRAIP expression and clinicopathologic features. Differences were considered statistically significant at *P* < 0.05 or *P* < 0.01.

## Results

### Overexpression of TRAIP in TNBC tissues and cells

**Fig. 1:**
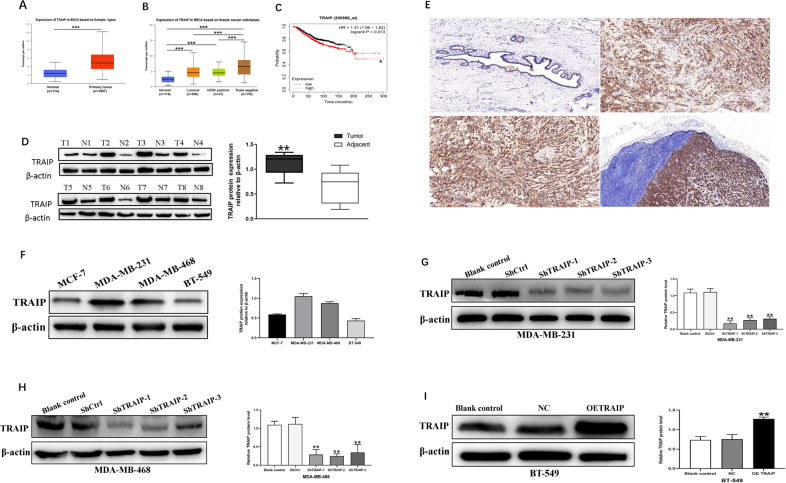
TRAIP expression levels in TNBC tissues and cells. **A**, **B** Comparison of TRAIP mRNA expression between normal tissue and primary tumor, and breast cancer subclasses. **C** Kaplan–Meier survival curves of 95 patients with high and low expression of TRAIP in TNBC. **D** TRAIP expressions were higher in TNBC than adjacent tissues at protein levels. T: tumor, A: adjacent tissue. **E** Immunohistochemistry showing TRAIP was higher in TNBC than in corresponding adjacent tissues. Moreover, the lymph node metastasis foci showed stronger TRAIP expression than the corresponding primary foci. Upper left: adjacent tissue, upper right: TNBC tissue, lower left: primary foci, lower right: lymph node metastasis foci. DAB (brown) served as chromogen. **F** The TRAIP protein levels in MCF-7, MDA-MB-231, MDA-MB-468 and BT-549 cells lines. **G**–**I** The silence and overexpression efficiency of TRAIP were detected by Western blot and β-actin were used as control. Data were expressed as the gray-scale ratio of TRAIP protein relative to that of β-actin. All data are shown as the means ± SD of three experiments. (***P < 0.001, **P < 0.01, *P < 0.05).

The UALCAN database was used to analyze the expression of TRAIP in breast cancer. The results showed that TRAIP was expressed higher in primary breast tumors (*n* = 1097) than in normal tissues (*n* = 114, Fig. 1A, *P* < 0.001). TRAIP expression increased in TNBC compared with luminal or HER2 + type (Fig. 1B, *P* < 0.001). In addition, The Kaplan–Meier plotter database showed that high TRAIP expression was significantly associated with poor prognosis of the 95 patients with TNBC (Fig. 1C, *P* < 0.05). Western blot assays were performed to detect the expression of TRAIP in 8 pairs of fresh TNBC tumors and adjacent tissues. The results showed that the protein levels (Fig. 1D, *P* < 0.01) of TRAIP in the tumor tissues were markedly higher than those in the corresponding adjacent tissues, suggesting TRAIP was overexpressed in TNBC. Furthermore, TRAIP expression was examined by immunohistochemistry and confirmed to be higher in breast cancer (Fig. 1E, upper right) than in the corresponding adjacent tissues (Fig. 1E, upper left, *P* < 0.01; Wilcoxon’s test; Table 1). Moreover, the TRAIP expression in the metastatic cancer tissues in the lymph node (Fig. 1E, lower right) was significantly higher than that in the primary cancer tissues (Fig. 1E, lower left, *P* < 0.01; Wilcoxon test; Table 1). In addition, patients with recurrence of carcinoma had higher TRAIP expression than those without recurrence (*P* < 0.05, Table 1). No significant differences were found between groups for age, tumor size, grade, or vascular invasion (Table 1).

The TRAIP protein levels in MCF-7, MDA-MB-231, MDA-MB-468, and BT-549 cells lines were evaluated using Western blot. The results showed that the TRAIP protein levels were significantly higher in MDA-MB-231 and MDA-MB-468 cell lines than in MCF-7 and BT-549 cell lines (Fig. 1F). Therefore, MDA-MB-231 and MDA-MB-468 were chosen for silencing TRAIP expression by using TRAIP-ShRNA (ShTRAIP-1/2/3). ShTRAIP-1/2 was more effective and thus used for the subsequent cell function experiments. BT-549 was used for overexpressing TRAIP (OETRAIP). Western blot analysis revealed that TRAIP expression was obviously silenced or overexpressed (Fig. 1G–I, *P* < 0.01).

### Silencing of TRAIP suppressed tumor cells proliferation and migration/invasion in vitro

**Fig. 2:**
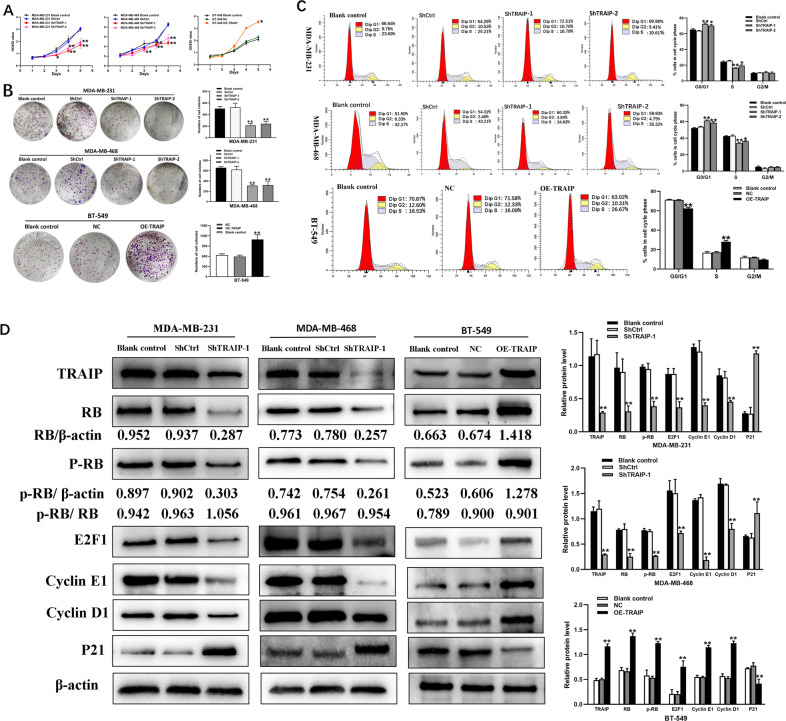
Silencing of TRAIP suppresses tumor cells proliferation and induces G1/S arrest though RB-E2F signaling. **A** Cell proliferation was examined 1, 2, 3, 4, and 5 days via CCK-8 assay. **B** Colony formation of TRAIP knockdown and overexpression group cells were photographed and colony numbers were illustrated in histogram. **C** Flow cytometry revealed the distribution of cell phase in the TNBC cell lines. **D** The expression of RB, phospho-RB, E2F1, Cyclin D1, CyclinE1 and P21 was examined by Western blot. All data are shown as the means ± SD of three experiments. (**P < 0.01, *P < 0.05).

CCK-8 and colony-formation assays were performed to determine the effect of TRAIP depletion on the proliferation in cancer cells. The results showed that the proliferation of MDA-MB-231 and MDA-MB-468 cells in the ShTRAIP-1/2 group was significantly inhibited compared with that of the control group (Fig. 2A, B, *P* < 0.01). Meanwhile, the growth of the OETRAIP group was significantly faster than that of the NC group in BT-549 cells. Given that cell proliferation was inhibited by TRAIP downregulation, the cell cycle distribution was examined by flow cytometry analysis. The G1 phase cells in the ShTRAIP-1/2 group showed significant enrichment, but the S phase cells were reduced relative to the ShCtrl group. However, an opposite result was observed in BT-549 cell (Fig. 2C, *P* < 0.01).

The expression of RB, phospho-RB, E2F1, Cyclin D1, CyclinE1, and P21 was examined by Western blot to explore the mechanism of TRAIP on the cell proliferation and cell cycle. The results showed that the expression of RB, phospho-RB, E2F1, CyclinD1, and CyclinE1 decreased and that of P21 increased in the ShTRAIP-1 group compared with those in the ShCtrl group of MDA-MB-231 and MDA-MB-468 cells. On the contrary, the expression of RB, phospho-RB, E2F1, CyclinD1, and CyclinE1 increased and that of P21 decreased in the OETRAIP group of BT-549 cells (Fig. 2D, *P* < 0.05 or 0.01). These results further suggested that silencing of TRAIP may induce G1/S arrest by repressing the RB-E2F signaling pathway.

**Fig. 3:**
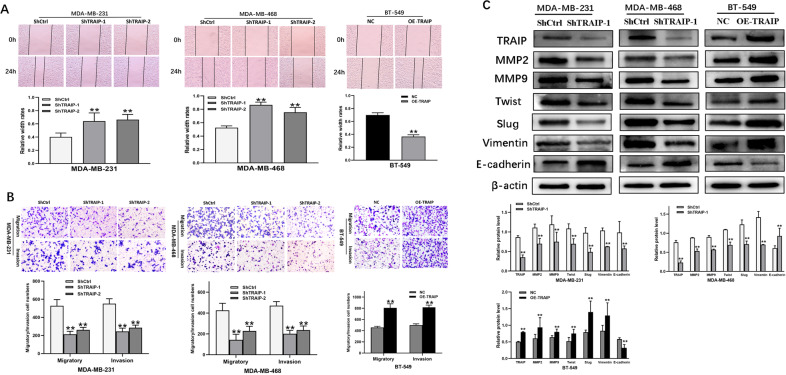
TRAIP knockdown inhibits TNBC cell lines migration and invasion by EMT. **A** The effect of TRAIP knockdown or overexpression on cell migration was determined by wound healing assay. **B** Transwell assays showed migration and invasion of MDA-MB-231, MDA-MB-468 and BT-549 cells. **C** Western blot were performed to detect the expression of MMP-2, MMP-9, Twist, Slug, Vimentin, E-cadherin. All data are shown as the means ± SD of three experiments. (**>*P* < 0.01, *P < 0.05).

Transwell assay and scratch test were performed to determine the effect of TRAIP gene knockdown on cell migration/invasion. The results of scratch test displayed that the ability of horizontal migration capacity was weakened compared with that in the control group of MDA-MB-231 and MDA-MB-468 cells (Fig. 3A, *P* < 0.01). Similarly, the vertical migratory and invasive capacity in the ShTRAIP-1/2 groups receded relative to those in the control group (Fig. 3B, *P* < 0.01). For confirmation of the above results, migration and invasion assays were also performed in the OETRAIP group (Fig. 3A, B, *P* < 0.01). All the experiments demonstrated that the cell’s migratory/invasive ability was progressively suppressed by TRAIP depletion.

The expression levels of MMP-2, MMP-9, Twist, Slug, Vimentin, and E-cadherin were detected by Western blot to further probe the effect of TRAIP in EMT. The results showed that the expression of E-cadherin increased and that of MMP-2, MMP-9, Twist, Slug, Vimentin decreased compared with those in the ShCtrl group of MDA-MB-231 and MDA-MB-468 cells. However, the expression of MMP-2, MMP-9, Twist, Slug, Vimentin increased and E-cadherin decreased in the OETRAIP group (Fig. 3C, *P* < 0.05 or 0.01). Consequently, these findings suggested that silencing of TRAIP may inhibit cell migration and invasion by suppressing EMT in breast cancer cells.

### MiR-590-3p directly targeted TRAIP and repressed TRAIP expression

**Fig. 4:**
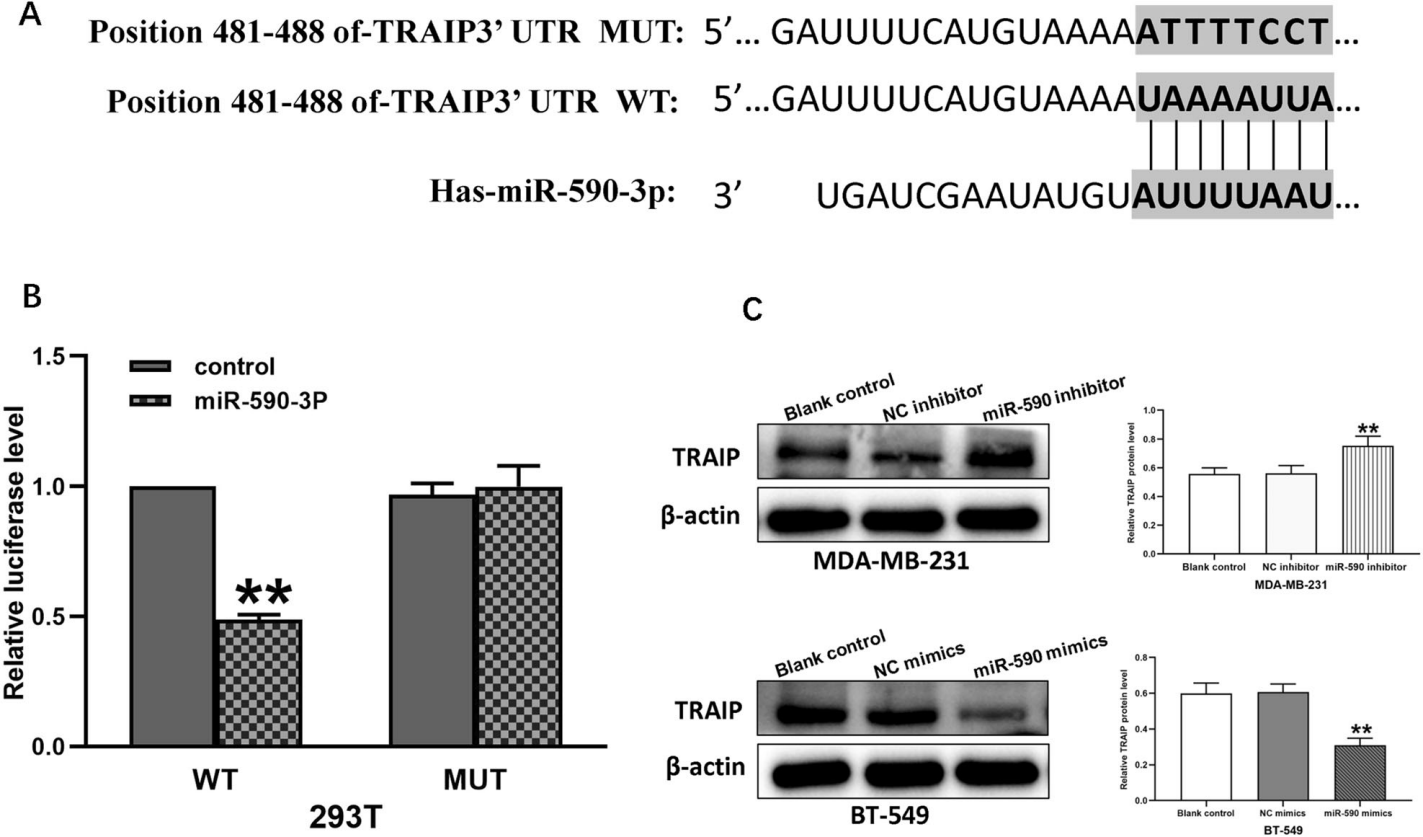
miR-590-3p directly targets TRAIP and represses TRAIP expression. **A** The binding sites of miR590-3p and TRAIP 3′-UTR. **B** Luciferase analysis showed the inhibitory effect of miR-590-3p on expression of the luciferase reporter gene by binding TRAIP 3′-UTR. **C** Western blot showed the TRAIP protein was highly up-regulated in miR-590-3p inhibitor and down-regulated by miR-590-3p mimic. All data are shown as the means ± SD of three experiments. (**P < 0.01).

By using online bioinformatics assay (Miranda, targetscan), the miRNA-targeting sites of miR590-3p on the 3′-UTR of TRAIP were found (Fig. 4A). In the luciferase reporter assays, the overexpression of miR-590-3p significantly reduced the luciferase activities of the WT TRAIP 3′-UTR reporter compared with the control. By contrast, when MUT sequence occurred at the binding sites, the luciferase level of the MUT UTR group showed no significant difference from the control group (Fig. 4B, *P* < 0.01). Therefore, TRAIP was a direct target of miR-590-3p. MiR-590-3p mimic (miR-590 mimic) and miR-590-3p inhibitor (miR-590 inhibitor) were transfected into MDA-MB-231 and BT-549 cells, respectively. Western blot assays indicated that miR-590-3p mimic could downregulate TRAIP expression, and miR-590-3p inhibitor could upregulate it (Fig. 4C, *P* < 0.01).

### Overexpression of miR-590-3p could restrain TNBC cell proliferation and migration/invasion

**Fig. 5:**
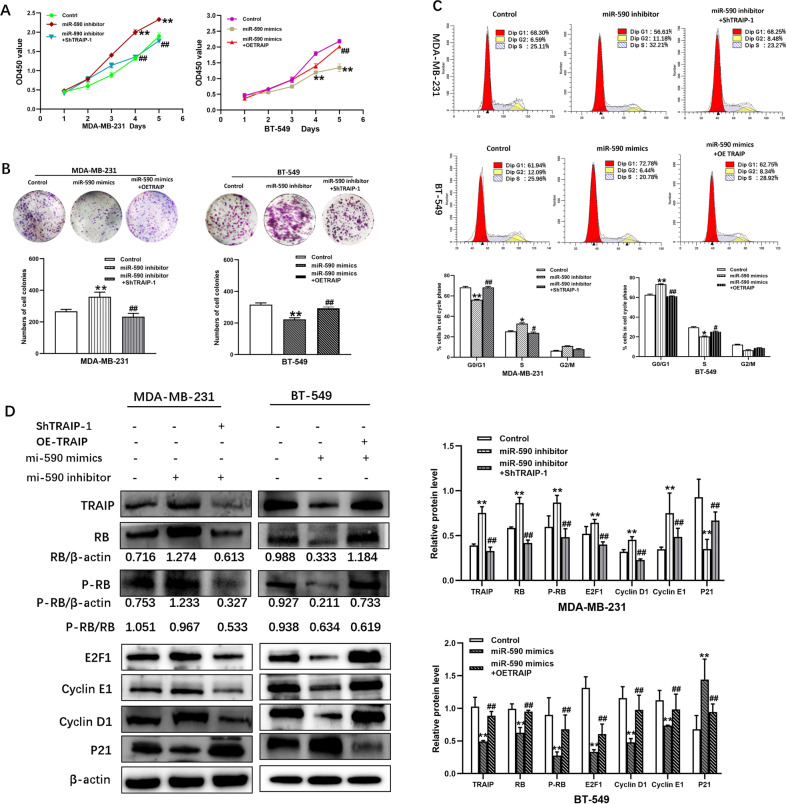
TRAIP reverses the effects of miR-590-3p on cells proliferation and induces G1/S arrest via RB-E2F signaling. **A** CCK-8 assay was examined 1, 2, 3, 4, and 5 days in MDA-MB-231 (Control group, miR-590 inhibitor group, miR-590 inhibitor+ShTRAIP-1 group) and BT-549 (Control group, miR-590 mimic group, miR-590 mimic + OETRAIP group). **B** Colony formation were photographed and colony numbers were illustrated in histogram. **C** Flow cytometry revealed the distribution of cell phase in MDA-MB-231 and BT-549 cell lines. **D** The expression of RB, phospho-RB, E2F1, CyclinE1, Cyclin D1 and P21 was examined by Western blot. All data are shown as the means ± SD of three experiments. (**P < 0.01, *P < 0.05, Control vs. miR-590-3p inhibitor, or Control vs. miR-590-3p mimic; ^**##**^*P* < 0.01, ^**#**^*P* < 0.05, miR-590 inhibitor vs. miR-590 inhibitor+ShTRAIP-1, or miR-590 mimic vs. miR-590 mimic+OETRAIP).

CCK-8 and colony-formation assays were performed to explore the effect of miR-590-3p mimic on the proliferation and migration/invasion in cancer cells. The results showed that the proliferation was markedly restrained in contrast to the NC mimic group (Fig. 5A, B, *P* < 0.01). Flow cytometry analysis revealed that the number of G1 phase cells was significantly enriched, whereas that of the S phase cells was reduced in the miR-590-3p mimic group (Fig. 5C, *P* < 0.05 or 0.01). Meanwhile, RB, Phospho-RB, E2F1, CyclinD1, and CyclinE1 decreased and P21 increased in the miR-590-3p mimic group compared with those in the NC mimic group of BT-549 (Fig. 5D, *P* < 0.05 or 0.01). This finding was also confirmed in the miR-590-3p inhibitor group of MDA-MB-231 cells. These results further suggested that overexpression of miR-590-3p induced G1/S arrest by repressing the RB-E2F signaling pathway.

**Fig. 6:**
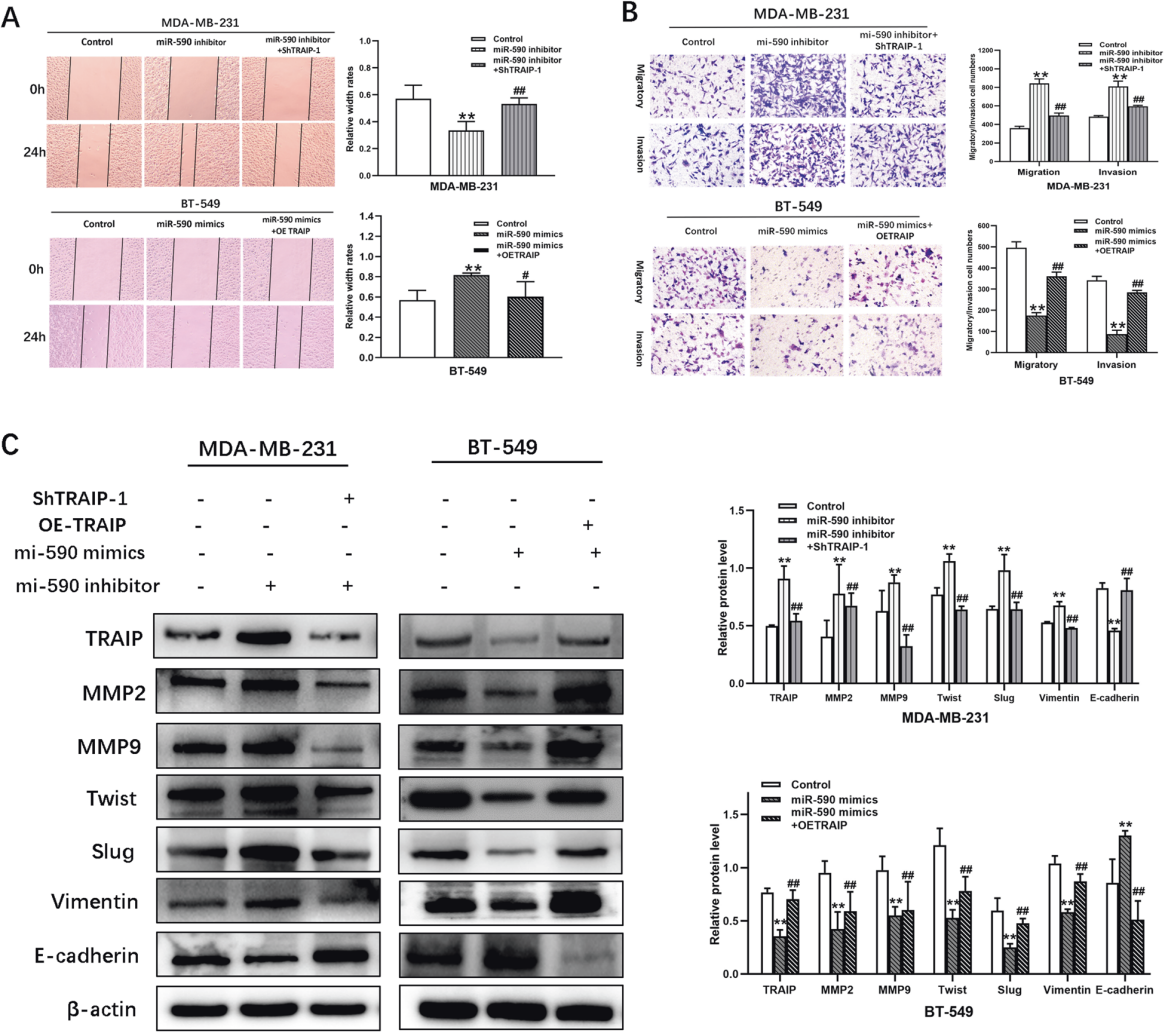
TRAIP reverses the effects of miR-590-3p on cells migration and invasion through EMT. **A** Wound healing assay was conducted to determine the effect between miR-590-3p and TRAIP on cell migration. **B** The migration and invasion of MDA-MB-231and BT-549 cells were showed by Transwell assays. **C** Western blot were performed to detect the expression of MMP-2, MMP-9, Twist, Slug, Vimentin, E-cadherin. All data are shown as the means ± SD of three experiments. MDA-MB-231 was transfected with miR-590 inhibitor, miR-590 inhibitor + ShTRAIP-1. BT-549 was transfected with miR-590 mimic, miR-590 mimic + OETRAIP. (**P < 0.01, *P < 0.05, Control vs. miR-590 inhibitor, or Control vs. miR-590 mimic; ^**##**^*P* < 0.01, ^**#**^*P* < 0.05, miR-590 inhibitor vs. miR-590 inhibitor + ShTRAIP-1, or miR-590 mimic vs. miR-590 mimic+OETRAIP).

The results of Transwell and scratch test displayed that the abilities of migration and invasion were restrained in the miR-590-3p mimic group of BT-549 cells. Similar results were found in MDA-MB-231 (Fig. 6A, B, *P* < 0.05 or 0.01). Consequently, the cell’s migratory/invasive ability was progressively suppressed by the overexpression of miR-590-3p. Some major EMT markers were detected by Western blot. The experimental results showed that the expression of E-cadherin increased and that of MMP-2, MMP-9, Twist, Slug, and Vimentin decreased compared with those in the NC mimic group of BT-549 cells. However, the expression of MMP-2, MMP-9, Twist, Slug, and Vimentin increased and that of E-cadherin decreased in the miR-590-3p inhibitor group (Fig. 6C, *P* < 0.05 or 0.01). Consequently, these findings suggested that the overexpression of miR-590-3p may inhibit cell migration and invasion by suppressing EMT in TNBC cells.

### Knockdown of TRAIP reversed the effect of miR-590-3p on proliferation, migration, and invasion in TNBC cells

A shift occurred when miR-590-3p inhibitor and LV3-hsa-TRAIP-318 (miR-590 inhibitor + ShTRAIP-1) were co-transfected into the MDA-MB-231 cells. In the rescue experiment, CCK-8 and colony-formation assays showed that the proliferation improvement caused by miR-590-3p inhibitor could be restored by silencing TRAIP (Fig. 5A, B, *P* < 0.01). Knockdown of TRAIP rescued the result that G1/S arrest caused by miR-590-3p inhibitor (Fig. 5C, *P* < 0.05 or 0.01). In RB-E2F signaling, RB, Phospho-RB, E2F1, CyclinD1, and CyclinE1 decreased and that of P21 increased in the miR-590 inhibitor + ShTRAIP-1 group compared with those in the miR-590 inhibitor group for MDA-MB-231 (Fig. 5D, *P* < 0.05 or 0.01). These results were also confirmed in BT-549 cells, which were co-transfected with miR-590-3p mimic and LV5-hsa-TRAIP-homo (miR-590 mimic + OETRAIP; Fig. 5A–D, *P* < 0.05 or 0.01). Therefore, silencing TRAIP may rescue the consequence that miR-590-3p inhibitor induced cell proliferation via the RB-E2F signaling pathway.

In addition, silencing of TRAIP reversed the results of Transwell and scratch tests, that is, the abilities of migration and invasion were reinforced by miR-590-3p inhibitor in MDA-MB-231 cells (Fig. 6A, B, *P* < 0.05 or 0.01). The expression of MMP-2, MMP-9, Twist, Slug, and Vimentin decreased and that of E-cadherin increased in miR-590 inhibitor + ShTRAIP-1 MDA-MB-231 cells, as confirmed by the miR-590 mimic + OETRAIP group (Fig. 6C, *P* < 0.05 or 0.01). These findings suggested that silencing TRAIP may reverse the effect of miR-590-3p inhibitor on the migratory and invasive abilities of TNBC cells through EMT.

### Silencing of TRAIP suppressed proliferation and metastasis in nude mice

**Fig. 7:**
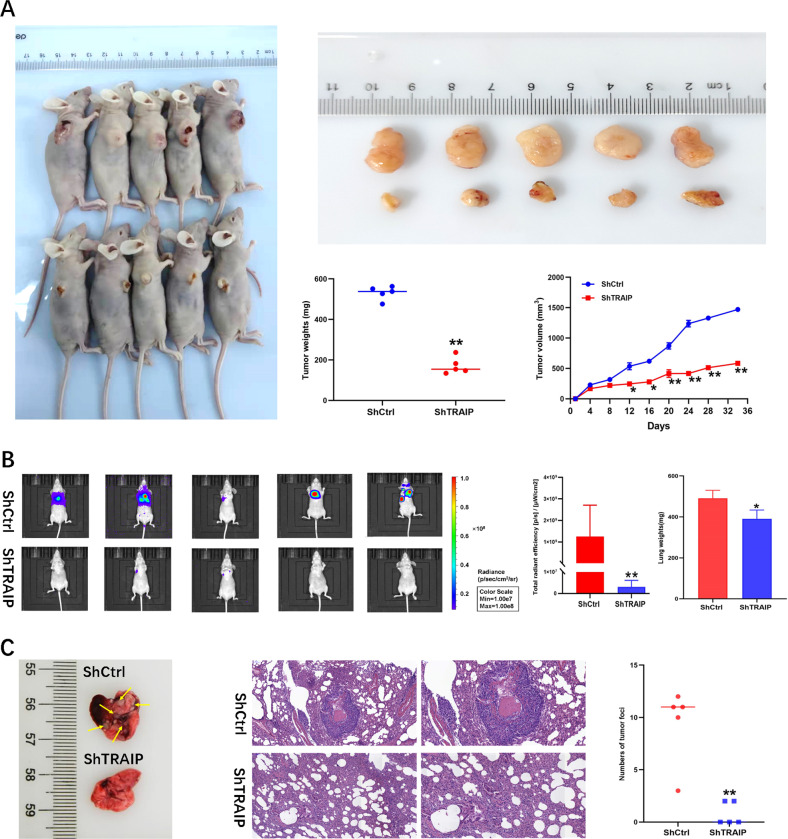
Silencing of TRAIP inhibits tumor growth and metastasis in nude mice. **A** ShCtrl and ShTRAIP-1 MDA-MB-231 cells were subcutaneously implanted into 5-week-old female BABL/c nude mice. The tumor volume was measured using the following formula: volume (mm^3^) = (width^2^×length)/2. **B** Six-week-old female nude BALB/c mice were injected with 1 × 10^6^ ShCtrl or ShTRAIP-1 MDA-MB-231 cells via the tail vein. Luciferase-signal and lung weight were exhibited. **C** Images of lung metastasis and H&E staining (left × 200; right ×400; upper: ShCtrl group; lower: ShTRAIP-1 group) are shown. (**P < 0.01, *P < 0.05).

Xenograft nude mouse models were established to further explore the growth and metastatic capacities of TRAIP in vivo. The results showed that the mouse tumors’ weight (533 ± 32 mg vs. 178 ± 34 mg) and tumor size (1430.2 ± 26.9 mm^3^ vs. 584.8 ± 12.28 mm^3^) in ShTRAIP-1 cell xenografts significantly decreased compared with those of the ShCtrl group (Fig. 7A, *P* < 0.05 or 0.01). Moreover, luciferase-labeled ShTRAIP-1 MDA-MB-231 cells were injected into female nude mice via the tail vein. After 40 days, only two out of the five mice in the ShTRAIP-1 group developed lung metastasis, whereas all five mice presented lung metastasis in the control group. The weights of lungs from the ShTRAIP-1 group were significantly lighter than those from the control group (Fig. 7B, *P* < 0.05 or 0.01). The number of tumor foci in the control group was much more than that in the ShTRAIP-1 group (Fig. 7C, *P* < 0.01). These results suggested that silencing of TRAIP could significantly inhibit TNBC proliferation and metastasis in vivo.

## Discussion

The tumor necrosis factor (TNF) superfamily contains various signaling proteins that regulate cell activity by binding to homologous cell receptors [32]. TNF receptor associated factor (TRAF) is a key scaffold connecting molecule in cells. Since the first TRAFs were cloned in the mid-1990s, TRAFs has made remarkable progress in regulating cell fate and cell death/survival [33]. Researchers used the yeast two-hybrid system to search for additional TRAF1-interacting proteins. Analysis of the DNA sequence of the TRAF1- and TRAF2-interacting cDNA clones revealed that they were derived from a single novel gene named TRAIP. In addition, TRAIP could interact directly with TRAF1 and TRAF2 in human cells (293 T cells) [34]. TRAIP inhibits the activation of NF-κB signaling pathway by inhibiting TRAF2 expression [35]. The cancer genome atlas (TCGA) is one of the most abundant databases of tumor data types in the world [36]. It has 1097 breast cancer data samples, including 1094 RNAseq data and 106 paired data samples [37, 38]. TRAIP gene was screened out by filtering and standardizing the original data [39–41]. The UALCAN database also observed that TRAIP expression in TNBC was increased compared with that in luminal or HER2 + type. Meanwhile, Western blot assay revealed that TRAIP was highly expressed in TNBC tissues and cells. Therefore, this study mainly focused on the role of TRAIP in TNBC.

TRAIP has carcinogenic properties, and it is negatively correlated with the prognosis of patients with liver cancer and lung cancer, suggesting that TRAIP may be a promising therapeutic target for those types of cancer [42, 43]. However, the expression and clinical significance of TRAIP in TNBC have not been verified. In the present study, the expression of TRAIP in TNBC was found to be higher than that in adjacent normal tissues for the first time, and the expression in lymph node metastasis was significantly higher than that in primary lymph node metastasis. Moreover, the high expression of TRAIP was significantly correlated with tumor recurrence, suggesting that the expression of TRAIP was significantly correlated with the progression of TNBC.

Previous studies have reported that knockdown of TRAIP in human epidermal keratinocytes inhibited cell proliferation and induced the G1/S phase arrest of cell cycle [44]. This finding is consistent with the results of cell proliferation experiment and xenograft assay in the present study, that is, the silencing of TRAIP could inhibit the proliferation of TNBC cells in vitro and in vivo and induce G1/S phase arrest. Chapard proved that TRAIP was a novel E2F target [45]. Phosphorylation and dephosphorylation of RB determined the activity of transcription factor E2F [46, 47]. Phosphorylation of RB caused the release of E2F1 and activated CyclinD1 and CyclinE1, which then initiated DNA replication and led to cell growth arrest [48, 49]. Cyclin-dependent kinase inhibitor p21 is one of the factors promoting cell cycle arrest under various stimuli [50]. In the present experiment, Western blot results showed that the expression of RB, Phospho-RB, E2F1, CyclinD1, and CyclinE1 decreased in the ShtrAIP-1 group, whereas that of P21 increased. Therefore, TRAIP was hypothesized to possibly promote TNBC cell proliferation and induce G1/S block through the RB-E2F signaling pathway, thus regulating cell proliferation and cycle distribution.

Overexpression of TRAIP could promote the migratory/invasive ability of osteosarcoma cells [51]. Transwell assays and nude mouse lung metastasis model in the present study showed that TRAIP could significantly promote the migratory and invasive ability of TNBC cells. Wei’s study showed that flag-TRAIP could co-precipitate with Myc-Twist1 [52]. Twist1 is well known to be one of the important transcription factors in the EMT pathway [53, 54]. E-cadherin is an epithelial adhesion that exists in human epithelial cells, and it is used to connect cells and transmit intracellular signals [55, 56]. Downregulation of E-cadherin could reduce adhesion between cells, which is conducive to metastasis and diffusion of malignant tumor cells [57, 58]. Snail could inhibit the transcription of E-cadherin, thus accelerating intercellular loosening and promoting the proliferation and metastasis of malignant tumor cells [59]. Vimentin could promote mesenchymal cell mobility in epithelial tumor cells [60]. The present study found that in the ShTRAIP-1 group, the expression levels of Twist, Slug, Vimentin, MMP-2, and MMP-9 significantly decreased and that of E-cadherin increased, whereas the OETRAIP group showed the opposite. Therefore, TRAIP may promote the migration and invasion of TNBC cells through the EMT pathway.

In addition, miR-590-3p was found to be one of the putative targets of TRAIP by using online bioinformatics assay. Our luciferase reporter assay showed TRAIP was a direct target of miR-590-3p. Recent studies reported that miR-590-3p acted as a tumor suppressor in glioblastoma multiform, medulloblastoma, hepatocellular carcinoma, and nephroblastoma [61]. In the present study, the proliferation and migration/invasion of TNBC cells were restrained by miR-590-3p mimic, which was similar to the results of other previous studies [62, 63]. Subsequently, the rescue experiment showed that the acceleration of proliferation and migration/invasion in TNBC cells caused by miR-590-3p inhibitor could be restored by silencing TRAIP.

**Fig. 8:**
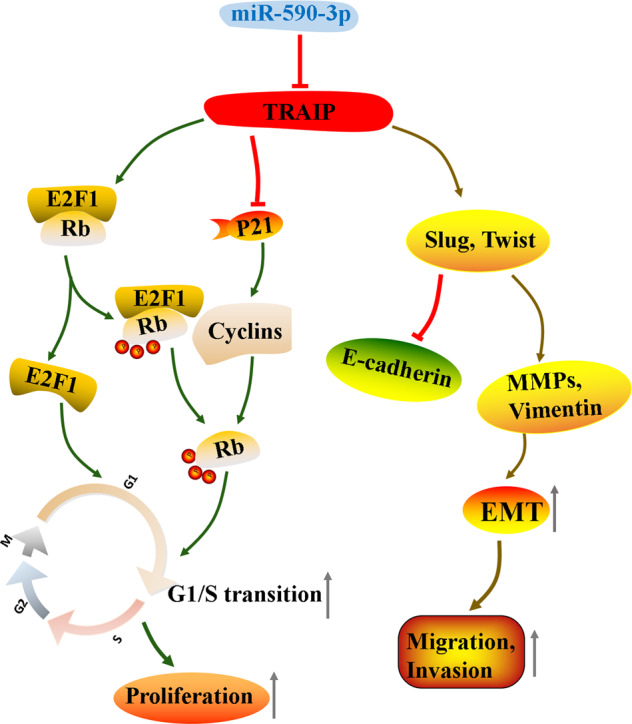
A model linking TRAIP to TNBC cell proliferation, migration and invasion via RB-E2F and SLUG/TWIST. Upon direct targeted by miR-590-3p, miR-590-3p has an opposite effect to TRAIP in TNBC cell lines, and TRAIP could reverse the results of miR-590-3p. TRAIP could facilitates G1/S transition and finally promotes TNBC cell proliferation. TRAIP also promotes processes of EMT, which finally accelerates TNBC cell migration and invasion.

Collectively, the results provided support, for the first time, that TRAIP was highly expressed in TNBC and miR-590-3p could directly target TRAIP. In addition, TRAIP knockdown may repress proliferation and migration/invasion by regulating RB-E2F signaling and EMT in TNBC cells (Fig. 8), suggesting that TRAIP plays a key regulatory role in cell proliferation and migration/invasion in TNBC.

## Data Availability

All remaining data and materials are available from the authors upon reasonable request.
